# First-Principles Calculations, Machine Learning and Monte Carlo Simulations of the Magnetic Coercivity of Fe_*x*_Co_1−*x*_ Bulks and Nanoclusters

**DOI:** 10.3390/nano15080577

**Published:** 2025-04-10

**Authors:** Dou Du, Youwei Zhang, Xingwu Li, Namin Xiao

**Affiliations:** AECC Beijing Institute of Aeronautical Materials, Beijing 100095, China; doudu_aecc@126.com (D.D.); ywzhang_pku@163.com (Y.Z.)

**Keywords:** FexCo1-x nanoclusters, density-functional theory, machine learning, Metropolis Monte Carlo, magnetic coercivity

## Abstract

FeCo alloys, renowned for their exceptional magnetic properties, such as high saturation magnetization and elevated Curie temperatures, hold significant potential for various technological applications. This study combines density-functional theory (DFT) and Monte Carlo (MC) simulations to investigate the magnetic properties of FeCo alloys and nanoclusters. DFT-derived exchange coupling constants ($J_{ij}$) and magnetic anisotropy ($K_{i}$) along with machine learning (ML) predicted spin vectors ($S_{i}$) serve as inputs for the Monte Carlo framework, enabling a detailed exploration of magnetic coercivity ($H_{c}$) across different compositions and temperatures. The simulations reveal an optimal Fe concentration, particularly around Fe_0.65_Co_0.35_, where magnetic coercivity reaches its peak, aligning with experimental trends. A similar simulation procedure was conducted for a Fe_58_Co_32_ nanocluster at 300 K and 500 K, demonstrating magnetic behavior comparable to bulk materials. This integrative computational approach provides a powerful tool for simulating and understanding the magnetic properties of alloys and nanomaterials, thus aiding in the design of advanced magnetic materials.

## 1. Introduction

Iron (Fe) and cobalt (Co) are transition metals renowned for their strong magnetic properties, making them essential components in various technological and industrial applications [1]. The electronic configurations of iron and cobalt atoms are [Ar] 3d^6^4s^2^ and [Ar] 3d^7^4s^2^, respectively. The presence of unpaired 3d electrons renders both iron and cobalt ferromagnetic. Pure iron exhibits a high saturation magnetization ($M_{s}$) of 2.2 $\mu_{B}$ per Fe atom and significant magnetic permeability at room temperature [2]. Pure cobalt, however, has the highest Curie temperature ($T_{c}$) among metals, at 1115 °C, compared to iron’s $T_{c}$ of 770 °C [3].

The combination of iron and cobalt elements forms the Fe_*x*_Co_1−*x*_ alloy, which exhibits remarkable magnetic properties [1,4,5,6]. Fe_*x*_Co_1−*x*_ nanoparticles, in particular, have gained attention for their biomedical applications due to their high magnetic anisotropy and moderate saturation magnetization, enabling efficient heat generation in cancer therapy through magnetic hyperthermia [7]. Furthermore, these nanoparticles enhance MRI imaging by providing superior contrast agent relaxivities and show promise in drug delivery systems, where their ability to bind and transport drugs to specific target sites improves therapeutic precision [8].

With advancements in modeling methods and high-performance computing (HPC), simulation techniques have become increasingly common for studying magnetic materials [9]. Electronic structure calculation methods, such as density-functional theory (DFT) [10], provide accurate descriptions of electronic spins, including magnetic moment values and orientations for individual atoms. However, DFT calculations are computationally expensive, particularly for large and complex systems. Moreover, capturing the dynamical evolution of a system’s magnetic state poses significant challenges when relying solely on electronic structure calculations. Researchers typically combine electronic structure calculations with micromagnetic simulations as a multiscale modeling approach to study magnetic materials [11,12,13,14,15,16,17]. At the electronic structure level, density-functional theory is used to calculate total energies for different spin configurations of the materials. A Heisenberg model-based Hamiltonian [18] is then constructed to derive magnetic parameters, primarily the exchange coupling constant ($J_{ij}$) and the magnetic anisotropy constant ($K_{i}$). These parameters serve as input values for micromagnetic simulations, which are performed using Monte Carlo (MC) or spin dynamics simulations [19].

## 2. Computational Methods

In this study, we used the plane-wave code PWmat [20,21] to perform all electronic structure calculations. Both Fe and Co possess 3*d* electrons and belong to the class of strongly correlated systems. Typically, the generalized gradient approximation (GGA) [22] struggles to accurately describe the localization of *d* and *f* electrons. Incorporating the Hubbard *U* term $E_{u}$ into the Kohn-Sham equations enhances the description of these *d* and *f* electrons, thus addressing the limitations of GGA [23]. In the simplified Dudarev approach [24], only the effective parameter $U_{eff}$ is used to compute the $E_{u}$ as shown in Equation (1). In Fe_*x*_Co_1−*x*_ systems, only the 3*d* electrons require correction via the Hubbard *U* term.

(1) $$E_{U}=\frac{U_{\mathrm{eff}}}{2}\sum_{\sigma }\sum_{m}\left(n_{mm}^{\sigma }-\sum_{m^{\prime }}n_{mm^{\prime }}^{\sigma }n_{m^{\prime }m}^{\sigma }\right)$$

There is no universal Hubbard *U* value applicable to all systems and properties. In this work, we aim to determine an appropriate *U* value for 3*d* electrons by fitting it to the Fe_*x*_Co_1−*x*_ structure and its magnetic properties, specifically the magnetic moments. To achieve this, we varied the *U* value for 3*d* electrons from 0 to 5 eV for both the body-centered cubic (BCC) Fe system and the hexagonal close-packed (HCP) Co system. All DFT calculations were performed using the plane-wave code PWmat. For the bulk structures, both the ionic positions and lattice parameters were optimized until the force on each atom was less than 0.01 eV/Å. The k-point grid was set to 11×11×11. Spin polarization was enabled using collinear spin configurations, without including contributions from spin-orbit coupling (SOC).

We construct the Hamiltonian for the Fe_*x*_Co_1−*x*_ systems based on the Heisenberg model [25], as shown in Equation (2). When preparing the input files for the DFT calculations, we initialize the spin orientations of atoms in Fe_*x*_Co_1−*x*_ randomly in a collinear configuration, allowing only spin-up (↑) or spin-down (↓) states. From the DFT calculations, we obtain the magnetic moments for each atom and the total energy of the system. By neglecting the orbital contribution, the spin vector $S_{i}$ can be derived from the magnetic moment using $M_{i}\approx -2\mu_{B}S_{i}/\hbar$.

By generating numerous Fe_*x*_Co_1−*x*_ supercells (4×4×4 unitcells) with randomized spin configurations and performing DFT calculations, we produced extensive data on atoms’ magnetic moments and total energies of the systems. Initially, we applied linear regression to fit Equation (2), determining the exchange coupling constants $J_{ij}$ and the magnetic anisotropy constant $K_{i}$. Subsequently, by leveraging the Fe_*x*_Co_1−*x*_ geometric structures and the calculated spin vectors $S_{i}$, we developed a machine learning model to predict the spin vectors $S_{i}$ for any given Fe_*x*_Co_1−*x*_ structure. A separate manuscript is in preparation to provide a detailed description of our machine learning approach, which is not discussed in detail in this paper.

(2) $$\begin{array}{cc} \hat{H}=E_{0}\; & -\frac{1}{2}J_{FeFe}\sum_{i\neq j}S_{i}^{Fe}S_{j}^{Fe}-\frac{1}{2}J_{FeCo}\sum_{i\neq j}S_{i}^{Co}S_{j}^{Fe}-\frac{1}{2}J_{CoCo}\sum_{i\neq j}S_{i}^{Co}S_{j}^{Co}\\ \; & +K_{Fe}\sum_{i}{\left(S_{i}^{Fe}\right)}^{2}+K_{Co}\sum_{i}{\left(S_{i}^{Co}\right)}^{2} \end{array}$$

Here, we mainly focus on the magnetic coercivity $H_{c}$ of the Fe_*x*_Co_1−*x*_ bulks and nanoclusters. The magnetic coercivity of a material is typically measured using a hysteresis loop tracer or vibrating sample magnetometer (VSM). A magnetic field is applied to the sample, and its magnetization is recorded as the field is cycled from positive to negative saturation, creating a hysteresis loop. The coercivity is determined as the magnetic field at which the material’s net magnetization becomes zero during the reversal process. To mimic the VSM experiments, the interaction term between the external magnetic field and the magnetic moment must be incorporated into the Hamiltonian. This interaction is represented by the Zeeman energy, as defined in Equation (3), where μ denotes the magnetic moment and B represents the strength of the external magnetic field.

(3) $$H_{Zeeman}=-\mu \cdot B$$

We neglected the spin–orbit coupling for the Fe_*x*_Co_1−*x*_ system, as it has minimal impact on the magnetic moment and total energies. The magnetic moment is approximated as shown in Equation (4):

(4) μ=−γS

where γ is the gyromagnetic ratio, which is a constant that relates the magnetic moment to the angular momentum of the particle. For an electron, the gyromagnetic ratio γ is given by

(5) $$\gamma =\frac{g_{s}\mu_{B}}{\hbar }$$

where $g_{s}$ is the electron spin *g*-factor, $\mu_{B}$ is the Bohr magneton, and ℏ is the reduced Planck constant. By incorporating the Zeeman Hamiltonian into Equation (2), we derive the modified Heisenberg Hamiltonian, presented as Equation (6), for the Fe_*x*_Co_1−*x*_ system under the influence of an external magnetic field $H_{ext}$ [26].

(6) $$\begin{array}{cc} {\hat{H}}^{\prime }=E_{0}\; & -\frac{1}{2}J_{FeFe}\sum_{i\neq j}S_{i}^{Fe}S_{j}^{Fe}-\frac{1}{2}J_{FeCo}\sum_{i\neq j}S_{i}^{Co}S_{j}^{Fe}-\frac{1}{2}J_{CoCo}\sum_{i\neq j}S_{i}^{Co}S_{j}^{Co}\\ \; & +K_{Fe}\sum_{i}{\left(S_{i}^{Fe}\right)}^{2}+K_{Co}\sum_{i}{\left(S_{i}^{Co}\right)}^{2}\\ \; & +\frac{g_{s}\mu_{B}}{\hbar }H_{\mathrm{ext}}\sum_{i}S_{i} \end{array}$$

With this new Hamiltonian, we performed Metropolis Monte Carlo simulations on Fe_*x*_Co_1−*x*_ nanoclusters. First, the geometry of the clusters was optimized using molecular dynamics (MD) simulations at the desired temperature. Next, the Monte Carlo simulation was initiated by randomly flipping a selected spin vector. The energy difference, ΔE, was calculated using Equations (8) and (9). The constants $J_{ij}$ and $K_{i}$ were derived from DFT calculations, while the spin vectors $S_{i}$ were predicted using a machine learning model. The acceptance of the step $P_{accept}$ was determined based on:

(7) $$P_{\mathrm{accept}}=\min\left[1,\exp\left(-\frac{\Delta E}{k_{B}T}\right)\right]$$

**Figure 1:**
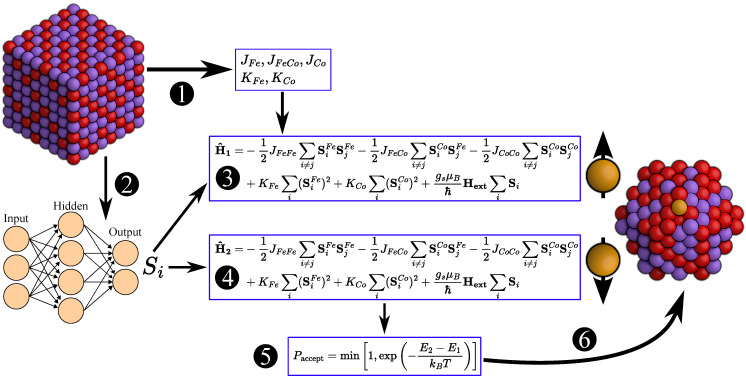
Schematic representation of the Monte Carlo simulation workflow for Fe_*x*_Co_1−*x*_ nanoclusters. Red atoms represent Fe, and purple atoms represent Co. This color scheme is used consistently throughout the paper. In step ➊, electronic structure calculations using DFT methods generate data to compute exchange coupling constants $J_{ij}$, anisotropy constants $K_{i}$ with linear regression. Using the DFT calculated atomic magnetic moment data to train a machine learning model for predicting the magnitude of the spin vectors $|S_{i}|$ (step ➋). The simulation begins with an MD-optimized Fe_*x*_Co_1−*x*_ alloys or nanoclusters at the desired temperature, calculating the initial energy $E_{1}$ using the Equation (8) shown in step ➌ with $J_{ij}$, $K_{i}$ and machine learning-predicted $S_{i}$. A random atom is selected, and its spin vector is flipped to compute the new energy $E_{2}$ with Equation (9) (step ➍). The change in energy ΔE is evaluated to decide whether to accept the spin vector update, following the Metropolis algorithm (step ➎). The nanocluster’s spin configuration is updated (step ➏), and steps ➌–➏ are repeated until the system converges to a stable energy minimum or reach the max number of steps.

A schematic of the Metropolis Monte Carlo simulation procedure is shown in Figure 1. In the Hamiltonian, we consider only interactions between nearest-neighbor atoms, as the exchange interaction decays rapidly with distance. Notably, when the spin vector of a single atom is flipped, the spin vectors of all atoms must be recomputed because our machine learning model predicts spin vectors based on the configurations of neighboring spins. This approach differs from traditional methods used in Monte Carlo simulations.

We developed Python code to perform Monte Carlo simulations of the Fe_*x*_Co_1−*x*_ bulk systems and nanoclusters. For the Fe_*x*_Co_1−*x*_ bulk systems, supercells were constructed with varying *x*-values, and the initial positions of iron and cobalt atoms were determined using a Monte Carlo annealing method. The resulting bulk structures were then optimized by molecular dynamics simulations at the desired temperature. Molecular dynamics simulations were conducted using the LAMMPS code [27] with the Modified Embedded Atom Method (MEAM) potential [28] within the NVT canonical ensemble. Choi et al. [28] demonstrated that the MEAM potential provides an accurate description of the Co-Fe system, effectively capturing lattice parameters, elastic properties, and thermodynamic properties. At each target temperature, the system achieved equilibrium within 1 ns, employing a time step of 1 fs. Periodic boundary conditions (PBCs) were applied to the bulk systems. The simulations for nanoclusters followed a similar procedure, but without the use of PBCs.

To simulate hysteresis loops, the Metropolis Monte Carlo simulations of the three-dimensional classical Heisenberg model involved applying a magnetic field that was incrementally varied, with the magnetization recorded to construct the hysteresis loop. Different algorithms, such as single spin flip (SSF) [29], cone algorithms, or global rotation cone algorithms [26], were utilized to ensure accurate and efficient modeling of collective spin dynamics, taking into account factors like anisotropy constants, temperature, and field scan speed. Here, we employed the SSF method to study the Fe_*x*_Co_1−*x*_ systems.

(8) $$\begin{array}{cc} {\hat{H}}_{1}=\; & -\frac{1}{2}J_{FeFe}\sum_{i\neq j}S_{i}^{Fe}S_{j}^{Fe}-\frac{1}{2}J_{FeCo}\sum_{i\neq j}S_{i}^{Co}S_{j}^{Fe}-\frac{1}{2}J_{CoCo}\sum_{i\neq j}S_{i}^{Co}S_{j}^{Co}\\ \; & +K_{Fe}\sum_{i}{\left(S_{i}^{Fe}\right)}^{2}+K_{Co}\sum_{i}{\left(S_{i}^{Co}\right)}^{2}+\frac{g_{s}\mu_{B}}{\hbar }H_{\mathrm{ext}}\sum_{i}S_{i} \end{array}$$

(9) $$\begin{array}{cc} {\hat{H}}_{2}=\; & -\frac{1}{2}J_{FeFe}\sum_{i\neq j}S_{i}^{Fe}S_{j}^{Fe}-\frac{1}{2}J_{FeCo}\sum_{i\neq j}S_{i}^{Co}S_{j}^{Fe}-\frac{1}{2}J_{CoCo}\sum_{i\neq j}S_{i}^{Co}S_{j}^{Co}\\ \; & +K_{Fe}\sum_{i}{\left(S_{i}^{Fe}\right)}^{2}+K_{Co}\sum_{i}{\left(S_{i}^{Co}\right)}^{2}+\frac{g_{s}\mu_{B}}{\hbar }H_{\mathrm{ext}}\sum_{i}S_{i} \end{array}$$

## 3. Results

This section details the calculated and simulated results. We begin with density-functional theory calculations for pure body-centered cubic iron and hexagonal close-packed cobalt, examining the influence of the Hubbard *U* parameter on their lattice parameters and magnetic moments. We then present Monte Carlo simulations of the magnetic coercivity of the Fe_*x*_Co_1−*x*_ bulks and nanocluster systems.

### 3.1. Density-Functional Theory Calculations

We specifically studied the effect of Hubbard *U* values on the calculated properties of pure iron and cobalt systems, with a focus on their lattice parameters and magnetic moments. The exchange interaction constant $J_{ij}$ is highly sensitive to the interatomic distances, while spin vectors $S_{i}$ are directly determined by magnetic moments. When the *U* value is set to zero, the density functional corresponds to PBE. We systematically increased the *U* value from 1 to 5 eV. By comparing the calculated lattice parameters and magnetic moments with experimental values, we aimed to identify the optimal Hubbard *U* value for accurately describing Fe_*x*_Co_1−*x*_ alloys. The calculated results and experimental values for BCC Fe and HCP Co are presented in Table 1 and Table 2, respectively.

Typically, the *U* values of iron and iron oxide systems are set between 3 and 5 eV [30,31], reflecting their electronic and magnetic properties. For cobalt systems, the appropriate *U* value varies depending on the specific compound and the oxidation state of Co. A review of various studies revealed that a reasonable range of values of *U* for cobalt lies between 3 and 7 eV [32,33,34]. These variations highlight the complexity of determining optimal *U* values, as they depend on the electronic structure, magnetic interactions, and experimental benchmarks of the material. However, in this study, we focus mainly on the lattice parameters and magnetic moments of the Fe_*x*_Co_1−*x*_ alloys. Table 1 and Table 2 clearly show that there does not exist a single *U* value with a good description of both the lattice parameters and magnetic moments of the pure Fe and Co systems. However, we can still clearly see that the PBE functional provides magnetic moments that are very close to the experimental data, and the lattice parameters are reasonably good. Furthermore, PBE is computationally much less expensive than the PBE+*U* method, reducing the overall computational cost. Thus, we employ the PBE functional for all electronic structure calculations in this study.

**Table 1:** The DFT + *U* calculated BCC Fe lattice parameter and magnetic moment per Fe atom.

Functional (*U* Value)	Lattice Parameter α (Å)	Magnetic Moment per Fe ($\mu_{B}$)
PBE	2.768	2.125
PBE + *U* (1 eV)	2.783	2.401
PBE + *U* (2 eV)	2.798	2.604
PBE + *U* (3 eV)	2.805	2.651
PBE + *U* (4 eV)	2.815	2.712
PBE + *U* (5 eV)	2.827	2.800
Expt. [35]	2.866	2.217

**Table 2:** The DFT + *U* calculated HCP Co lattice parameters and magnetic moment per Co atom.

Functional (*U* Value)	Lattice Parameter α (Å)	Lattice Parameter *c* (Å)	Magnetic Moment per Co ($\mu_{B}$)
PBE	2.408	3.906	1.532
PBE + *U* (1 eV)	2.408	3.911	1.584
PBE + *U* (2 eV)	2.407	3.915	1.631
PBE + *U* (3 eV)	2.405	3.922	1.668
PBE + *U* (4 eV)	2.395	3.998	1.773
PBE + *U* (5 eV)	2.420	3.876	1.721
Expt. [36]	2.507	4.069	1.58

### 3.2. Exchange Coupling $J_{ij}$, Anisotropy $K_{i}$, and Spin Vector $|S_{i}|$

**Figure 2:**
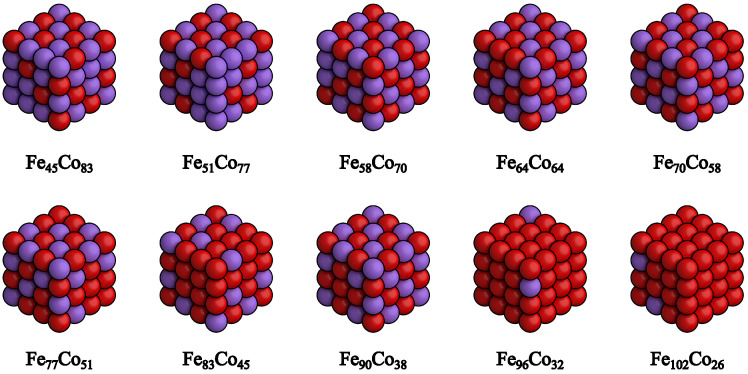
Body-centered cubic Fe_*x*_Co_1−*x*_ bulk structures were generated for a range of compositions. The iron concentration was varied from 35% in Fe_45_Co_83_ to 80% in Fe_102_Co_26_. To prepare these models for subsequent calculations, the atomic positions of iron and cobalt within the supercells were pre-optimized by using a Monte Carlo annealing method.

After opting to use the PBE functional to study Fe_*x*_Co_1−*x*_ systems, we generated a variety of compositions (with the structures shown in Figure 2) for Fe_*x*_Co_1−*x*_ with diverse configurations with randomly flipped spins. The compositional parameter *x* ranged from 35% in Fe_45_Co_83_ to 80% in Fe_102_Co_26_. According to the Fe-Co binary phase diagram [2], these compositions exhibit a BCC structure below 1000 K. The positions of the iron and cobalt atoms were pre-optimized by using a Monte Carlo annealing process. All Fe_*x*_Co_1−*x*_ structures adopted the BCC configuration, with the lattice parameters determined by linear interpolation between the calculated BCC structures of pure Fe and Co, based on the *x* value.

Subsequently, we performed DFT calculations on the Fe_*x*_Co_1−*x*_ structures, optimizing only the positions of the atoms to obtain the total energies $E_{\mathrm{tot}}$ and the magnetic moments of individual atoms. The spin vectors $S_{i}$ were calculated from the magnetic moments using Equation (4). By substituting all $E_{\mathrm{tot}}$ and $S_{i}$ values into Equation (2), we applied linear regression to fit the data and extracted the magnetic parameters $J_{ij}$ and $K_{i}$, as summarized in Table 3.

The magnetic anisotropy $K_{i}$ for both Fe and Co was found to be very small and can be considered negligible in our calculations, indicating minimal magnetic anisotropy in the BCC-structured Fe_*x*_Co_1−*x*_. We also determined the exchange coupling constants for Fe–Fe ($J_{\mathrm{FeFe}}$), Fe–Co ($J_{\mathrm{FeCo}}$), and Co–Co ($J_{\mathrm{CoCo}}$) interactions. The $J_{\mathrm{FeFe}}$ value was slightly larger, in agreement with previous studies; however, the differences among the three coupling constants were not substantial. In contrast to many studies that constrain $S_{i}$ to ±1, we employed the calculated values of $S_{i}$, which helped reduce the variability in the exchange coupling constants.

**Figure 3:**
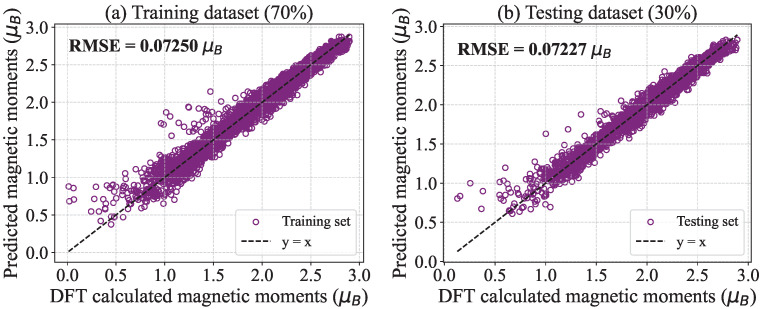
The training (**a**) and testing (**b**) datasets were prepared for a machine learning model designed to predict magnetic moments, specifically the magnitude of the spin vector $|S_{i}|$.

Concurrently, we employed a machine learning approach to develop a model for predicting the magnitude of the spin vector $|S_{i}|$ based on the calculated magnetic moment data. The dataset, consisting of 14,108 entries, was split such that 9885 entries (70%) were used for training the model, while 4223 entries (30%) were reserved for testing. The results are presented in Figure 3. Notably, the magnetic moment values exhibited a wide distribution, ranging from 0 to 3.0 $\mu_{B}$, indicating a strong correlation between an atom’s magnetic moment (μ) and its local environment. The small root-mean-square error (RMSE) values suggest that the machine learning model provides accurate predictions of the spin magnitude $|S_{i}|$.

### 3.3. Monte Carlo Simulations of the Fe_*x*_Co_1−*x*_ Bulks

Using the machine learning model trained on first-principles data, we predicted $S_{i}$ for given Fe_*x*_Co_1−*x*_ geometries. Following the procedure outlined in Figure 1, Metropolis Monte Carlo simulations were carried out on various Fe_*x*_Co_1−*x*_ bulk systems with different *x*-values. The positions of Fe and Co atoms within the Fe_*x*_Co_1−*x*_ lattices were initially optimized using the Monte Carlo annealing method, followed by further relaxation of the bulk systems through molecular dynamics simulations conducted at 300 K. The *x*-value ranged from 35% (Fe_45_Co_83_) to 80% (Fe_102_Co_26_). According to the experimental phase diagram, the Fe_*x*_Co_1−*x*_ alloy adopts a BCC structure within this composition range.

To simulate the hysteresis loops of magnetic materials, two primary Monte Carlo algorithms are commonly employed: the single-spin-flip (SSF) and cluster-spin-flip (CSF) algorithms, which differ in their spin update mechanisms. Our approach is based on the SSF algorithm; however, in our method, flipping a single spin alters the magnetic moments of its nearest and next-nearest neighbor spins, as predicted by the machine learning model. As a result, the magnetic moments of these neighboring spins must be recalculated to accurately reflect the influence of the spin flip.

**Figure 4:**
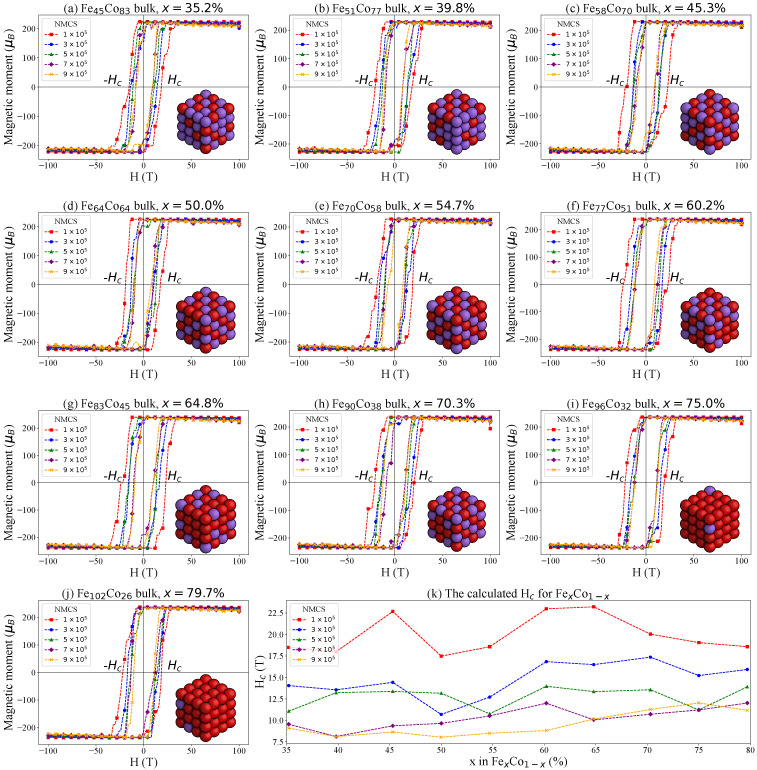
Simulated hysteresis loops for different Fe_*x*_Co_1−*x*_ bulk systems with varying numbers of Monte Carlo steps (NMCS) at 300 K: (**a**) Fe_45_Co_83_ (*x* = 35.2%), (**b**) Fe_51_Co_77_ (*x* = 39.8%), (**c**) Fe_58_Co_70_ (*x* = 45.3%), (**d**) Fe_64_Co_64_ (*x* = 50.0%), (**e**) Fe_70_Co_58_ (*x* = 54.7%), (**f**) Fe_77_Co_51_ (*x* = 60.2%), (**g**) Fe_83_Co_45_ (*x* = 64.8%), (**h**) Fe_90_Co_38_ (*x* = 70.3%), (**i**) Fe_96_Co_32_ (*x* = 75.0%), (**j**) Fe_102_Co_26_ (*x* = 79.7%) and (**k**) the calculated $H_{c}$ values for Fe_*x*_Co_1−*x*_.

We computed hysteresis loops for nine different compositions of Fe_*x*_Co_1−*x*_ using five distinct numbers of Monte Carlo steps (NMCS) at 300 K, ranging from $1\times 10^{5}$ to $9\times 10^{5}$. Each Monte Carlo step corresponds to a single attempt to flip a spin. The NMCS indicates the total number of Monte Carlo steps performed at a given temperature and under a fixed external magnetic field (*H*). Once the specified NMCS is reached, the total magnetic moment is recorded. Subsequently, the external magnetic field is incrementally increased (or decreased) by a small constant, and the Monte Carlo simulation is repeated. This iterative procedure yields a complete simulated hysteresis loop for each Fe_*x*_Co_1−*x*_ composition. The results are presented in Figure 4.

**Figure 5:**
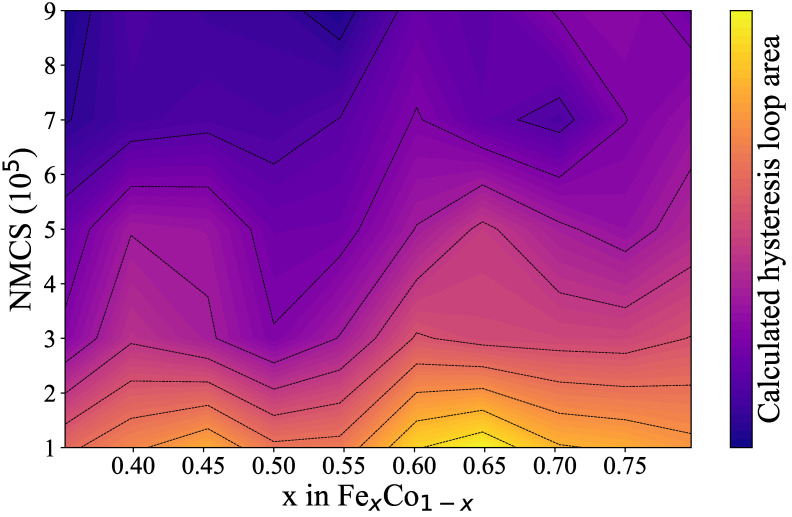
Calculated hysteresis loop areas were determined by varying the x in Fe_*x*_Co_1−*x*_ and the number of Monte Carlo steps (NMCS), visualized using a colormap.

The magnetic coercivity ($H_{c}$) for each hysteresis loop is determined as the external field value at which the net magnetic moment becomes zero. Additionally, we evaluated the hysteresis loss, which is directly proportional to the area enclosed by the hysteresis loop (see, e.g., Equation (10)), as it provides valuable insight into energy dissipation during magnetization and demagnetization processes. The area enclosed by each hysteresis loop was computed and visualized using a colormap, as shown in Figure 5.

(10) $$P_{h}=\oint HdB$$

The NMCS corresponds to the sweep rate or field ramp rate in experimental hysteresis loop measurements. At higher sweep rates (i.e., smaller NMCS values), the magnetic system has less time to respond to the changing external field, leading to non-equilibrium conditions in which domain wall motion and magnetization reversal lag behind the applied field. This lag necessitates a stronger reverse field to bring the net magnetization to zero, thereby increasing the measured coercivity. In contrast, at slower sweep rates (i.e., larger NMCS values), the system has more time to overcome energy barriers through thermal fluctuations. Consequently, domain walls and spins can more readily adapt to changes in the external field, resulting in lower coercivity values. Figure 4 clearly illustrates these trends across different NMCS values, showing that increasing the NMCS consistently leads to a reduction in $H_{c}$ for all Fe_*x*_Co_1−*x*_ bulk compositions.

By plotting the coercivity $H_{c}$ as a function of *x* in Fe_*x*_Co_1−*x*_, we observe two local maxima in the curve: one near 45% and another in the range of 60–65% (see Figure 4). Correspondingly, the colormap of integrated hysteresis loop areas reveals two regions with elevated values at 45% and 65%, which appear as bright regions in Figure 5. The composition of 65% Fe in Fe_*x*_Co_1−*x*_ (Fe_0.65_Co_0.35_) is widely recognized as a “magic number” for FeCo alloys. Numerous studies have shown that the Fe_0.65_Co_0.35_ composition exhibits the highest saturation magnetization among all Fe_*x*_Co_1−*x*_ alloys, making it particularly valuable for a range of technological applications [37,38,39,40,41,42,43,44,45,46,47,48,49].

**Figure 6:**
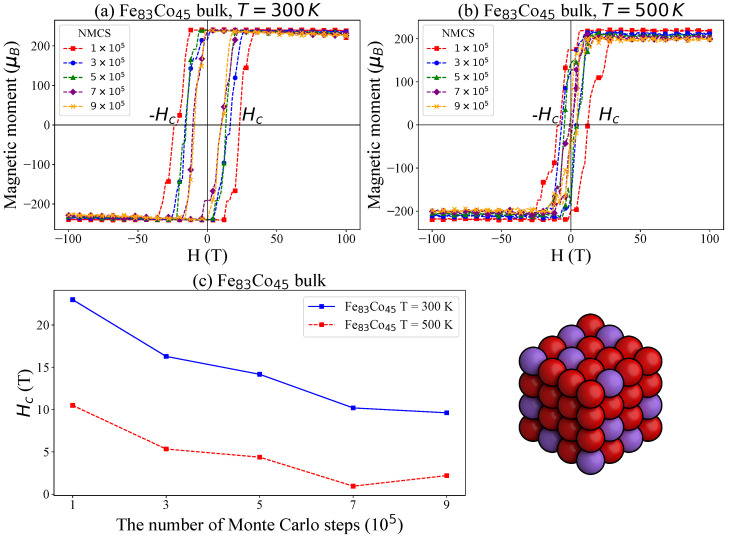
Simulated hysteresis loops for the Fe_83_Co_45_ bulk system with varying numbers of Monte Carlo steps (NMCS): (**a**) simulations at 300 K, (**b**) simulations at 500 K, and (**c**) the trend in magnetic coercivity with increasing NMCS.

Among the bulk samples studied, Fe_83_Co_45_ has an *x*-value (64.8%) closest to 65%. We further investigated the magnetic behavior of Fe_83_Co_45_ at an higher temperature of 500 K. The simulated hysteresis loops and the corresponding coercivity ($H_{c}$) values are presented in Figure 6, along with a comparison to the results obtained at 300 K. As shown, the hysteresis loops at 500 K are significantly narrower than those at 300 K, leading to consistently lower $H_{c}$ values across all examined NMCS. At higher temperatures, the increased thermal energy competes with the magnetic anisotropy energy that stabilizes the magnetization direction. This competition lowers the energy barriers for magnetization reversal, thereby facilitating the reorientation of magnetic moments and resulting in a reduced coercive field.

### 3.4. Monte Carlo Simulations of the Fe_58_Co_32_ Nanocluster

**Figure 7:**
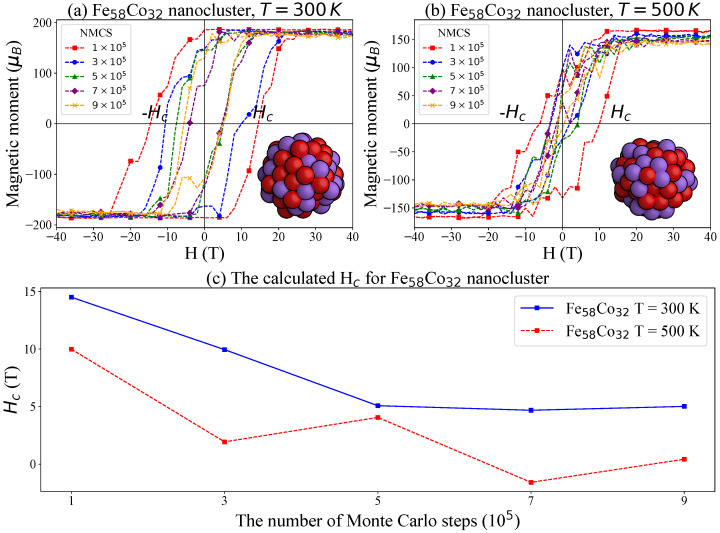
Simulated hysteresis loops for the Fe_58_Co_32_ nanocluster with varying numbers of Monte Carlo steps (NMCS): (**a**) simulations at 300 K, (**b**) simulations at 500 K, (**c**) the calculated $H_{c}$ values for Fe_58_Co_32_ nanocluster.

Furthermore, we investigated Fe_*x*_Co_1−*x*_ nanoclusters with an *x*-value close to 65%. To this end, we applied the same simulation procedure to the Fe_58_Co_32_ nanocluster, which has an *x*-value of 64.4%. Molecular dynamics simulations were used to optimize the structure at both 300 K and 500 K. The simulated hysteresis loops and corresponding coercivity ($H_{c}$) values are shown in Figure 7. At 300 K, the coercive field of the Fe_58_Co_32_ nanocluster is lower than that of the Fe_83_Co_45_ bulk system, which aligns with the experimental observations [50]. Nanoparticles typically exhibit smaller coercive fields as a result of the presence of fewer magnetic domains or even single-domain behavior [51]. At 500 K, the coercivity decreases further, indicating a strong influence of thermal fluctuations on magnetic stability at the nanoscale.

## 4. Discussion

The investigation of the magnetic properties of Fe_*x*_Co_1−*x*_ alloys and nanoclusters highlights the intricate interplay between the composition, temperature, and size effects. Using a combined approach of density functional theory, machine learning, and Monte Carlo simulations, the study underscores the potential of computational methods to accurately predict and optimize magnetic coercivity for advanced technological applications.

The composition of Fe_*x*_Co_1−*x*_ alloys plays a pivotal role in determining their magnetic coercivity. The simulations reveal distinct peaks in $H_{c}$ at specific Fe-Co ratios, notably around 45% and 65% Fe content. These results are consistent with experimental observations that identify optimal compositions for achieving enhanced magnetic performance. In particular, the “magic number” composition of Fe_0.65_Co_0.35_ exhibits the highest saturation magnetization, making it a prime candidate for applications requiring superior magnetic properties. The observed trends can be attributed to the sensitivity of the exchange coupling constants ($J_{ij}$) and magnetic moments to the local atomic configuration within the alloy, highlighting the critical influence of compositional tuning on magnetic behavior.

Temperature emerges as a critical factor influencing the magnetic behavior of both bulk and nanoscale systems. Elevated temperatures result in a pronounced reduction in $H_{c}$, driven by increased thermal energy that competes with the magnetic anisotropy energy. This thermal agitation promotes spin reorientation, effectively lowering the energy barriers for magnetization reversal. A comparative analysis between 300 K and 500 K clearly illustrates this trend, with significantly narrower hysteresis loops and reduced $H_{c}$ values observed at higher temperatures. These insights are particularly valuable for the design of magnetic materials intended for operation in high-temperature environments.

This study also highlights the pronounced differences in magnetic properties between bulk materials and nanoclusters. Nanoclusters, such as the Fe_58_Co_32_ system, exhibit a reduced $H_{c}$ compared to their bulk counterparts. This reduction is consistent with theoretical predictions, which attribute the lower coercivity in nanoclusters to finite-size effects, the presence of fewer magnetic domains, and enhanced thermal fluctuations. These findings underscore the importance of precise compositional control and size optimization in tailoring the magnetic properties of nanoscale materials for targeted applications.

The use of machine learning-augmented Metropolis Monte Carlo simulations provides a robust framework for modeling the dynamic behavior of spin systems. By incorporating parameters derived from DFT calculations, these simulations effectively capture the complex dependencies of $H_{c}$ on compositional and structural variables. The iterative approach, involving random spin vector flips and energy minimization, offers a detailed representation of hysteresis behavior across varying Fe-Co compositions and Monte Carlo steps. Notably, the observed decrease in $H_{c}$ with increasing Monte Carlo steps reflects the system’s progression toward equilibrium conditions, consistent with experimental observations of sweep rate effects in hysteresis measurements.

Our multiscale approach bridges the gap between theoretical predictions and experimental observations, offering a computationally efficient pathway for the design of advanced magnetic materials. The findings underscore the feasibility of optimizing magnetic coercivity through precise control over composition, temperature, and size. The demonstrated approach can be readily extended to other alloy systems, paving the way for the development of next-generation materials for applications in data storage, biomedical devices, and high-temperature technologies.

## 5. Conclusions

We successfully integrate first-principles calculations, machine learning, and Monte Carlo simulations to investigate the magnetic coercivity of Fe_*x*_Co_1−*x*_ alloys and nanoclusters. The results reveal that intermediate Fe concentrations, particularly around 65%, lead to maximum magnetic coercivity, highlighting the critical role of compositional tuning. These insights provide valuable guidelines for tailoring Fe-Co alloys for specific applications, especially in high-temperature environments and nanoscale systems. By demonstrating the synergy between computational techniques and machine learning, this work establishes a versatile framework for exploring and optimizing the magnetic properties of complex materials. The approach presented here provides a powerful tool for the rational design of next-generation magnetic materials for a broad range of technological applications.

## Figures and Tables

**Table 3:** Values of exchange coupling ($J_{ij}$) and anisotropy ($K_{i}$) determined by linear regression fitting.

Magnetic Parameters	Values
$J_{FeFe}$	0.010837 eV
$J_{FeCo}$	0.010061 eV
$J_{CoCo}$	0.010338 eV
$K_{Fe}$	≈0
$K_{Co}$	≈0

## Data Availability

The original contributions presented in this study are included in the article. Further inquiries can be directed to the corresponding authors.

## Abbreviations

The following abbreviations are used in this paper:

DFT	Density-Functional Theory
ML	Machine Learning
MC	Monte Carlo
HPC	High-Performance Computing
GGA	Generalized Gradient Approximation
BCC	Body-Centered Cubic
HCP	Hexagonal Close-Packed
SOC	Spin-Orbit Coupling
VSM	Vibrating Sampling Magnetometer
MD	Molecular Dynamics
MEAM	Modified Embedded Atom Method
PBCs	Periodic Boundary Conditions
SSF	Single Spin Flip
CSF	Cluster Spin Flip
RMSE	Root-Mean-Square Error
NMCS	Number of the Monte Carlo Steps
